# Molecular identification, transcript expression, and functional deorphanization of the adipokinetic hormone/corazonin-related peptide receptor in the disease vector, *Aedes aegypti*

**DOI:** 10.1038/s41598-018-20517-8

**Published:** 2018-02-01

**Authors:** Azizia Wahedi, Jean-Paul Paluzzi

**Affiliations:** Department of Biology, York University, Toronto, M3J 1P3 Ontario Canada

## Abstract

The recently discovered adipokinetic hormone/corazonin-related peptide (ACP) is an insect neuropeptide structurally intermediate between corazonin (CRZ) and adipokinetic (AKH) hormones, which all demonstrate homology to the vertebrate gonadotropin-releasing hormone (GnRH). To date, the function of the ACP signaling system remains unclear. In the present study, we molecularly identified the complete open reading frame encoding the *Aedes aegypti* ACP receptor (ACPR), which spans nine exons and undergoes alternative splicing giving rise to three transcript variants. Only a single variant, *Aedae*ACPR-I, yielding a deduced 577 residue protein, contains all seven transmembrane domains characteristic of rhodopsin-like G protein-coupled receptors. Functional deorphanization of *Aedae*ACPR-I using a heterologous cell culture-based system revealed highly-selective and dose-dependent receptor activation by *Aedae*ACP (EC_50_ = 10.25 nM). Analysis of the *Aedae*ACPR-I and *Aedae*ACP transcript levels in all post-embryonic developmental stages using quantitative RT-PCR identified enrichment of both transcripts after adult eclosion. Tissue-specific expression profiling in adult mosquitoes reveals expression of the *Aedae*ACPR-I receptor transcript in the central nervous system, including significant enrichment within the abdominal ganglia. Further, the *Aedae*ACP transcript is prominently detected within the brain and thoracic ganglia. Collectively, these results indicate a neuromodulator or neurotransmitter role for ACP and suggest this neuropeptide may function in regulation of post-ecdysis activities.

## Introduction

Neuropeptides are structurally and functionally the most diverse class of signaling molecules that function in intercellular communication in multicellular organisms^1^. In insects, neuropeptides play a fundamental role in the regulation of various physiological processes including development, reproduction, osmoregulation, as well as behavior and feeding^2^. One of the first neuropeptides to be isolated and purified from insects was the adipokinetic hormone (AKH), which is produced by neurosecretory cells of the corpora cardiaca (CC), and is one of the most extensively studied family of neuropeptides^3^. Insect AKH neuropeptides have been functionally well characterized to stimulate the release of energy-rich compounds including diacylglycerols, trehalose, and in some cases proline, into the haemolymph to fuel the activity of the insect^4^. In addition to these catabolic actions, AKH peptides have been shown to stimulate heart beat and inhibit the synthesis of haemolymph and tissue proteins^5^. The AKH receptors (AKHR), first isolated from *Drosophila melanogaster* and *Bombyx mori*, are G-protein coupled receptors (GPCR)^6,7^. Both AKH and AKHR demonstrate homology with the vertebrate gonadotropin releasing hormone (GnRH) and its receptor (GnRHR), respectively^6,8^.

Another insect neuropeptide family that is structurally similar to the vertebrate GnRH signaling system are the corazonin peptides. Corazonin (CRZ) is an insect undeca-neuropeptide first discovered in the cockroach, *Periplaneta americana*, due to its significant cardioexcitatory activity on the isolated cockroach heart^9^. A similar cardioexcitatory role has been established in other insects, including the kissing bug, *Rhodnius prolixus*^10^; however, this cardiostimulatory function is not universal since corazonin lacks cardiomyotropic activity in adult stage *Anopheles gambiae* mosquitoes^11^. Despite the CRZ sequence remaining largely conserved across insect species (primarily pQTFQYSRGWTNamide), to date no universal function has been described for this neuropeptide^12^. Nonetheless, multiple physiological roles have been described for CRZ, including the induction of melanization in swarming populations of *Locusta migratoria* and *Schistocerca gregaria*, initiation of ecdysis in *Manduca sexta*, the reduction in silk spinning rates in *B. mori*, and in social insects, CRZ has been proposed as a central regulator of behaviour and caste identity^13–16^.

Over the last decade, a third signaling system evolutionarily- and structurally-related to the AKHRs and CRZRs and their neuropeptides was identified and named adipokinetic hormone/corazonin-related peptide (ACP)^17^. ACP and its receptor (ACPR), like AKH/AKHR and CRZ/CRZR, also demonstrate homology to the vertebrate GnRH/GnRHR system. Upon analysis of this structural intermediate it became evident that ACP and its receptor ACPR were in fact already described in a number of insects, however, were at the time characterized as AKHs and AKHRs^18–24^. Unlike AKH, but similar to CRZ, the ACP amino acid sequence beginning from the N-terminus (pQVTFSRDW) demonstrates conservation across most arthropods^17^. Characterization of the ACP signaling system in *R. prolixus*, *Tribolium castaneum*, and *A. gambiae* revealed that the AKH, CRZ, and ACP receptors were only activated by their corresponding ligand, and thus are suggested to be independent signaling systems^17,25^. However, *in vitro* studies characterizing *B. mori* AKHR and ACPR have shown that high concentrations of ACP can activate the AKH receptor, and vice versa^22,23^. In both *R. prolixus* and *A. gambiae*, it was also shown that CRZ cannot activate either ACPR or AKHR and furthermore, neither AKH nor ACP can activate CRZR, which indicates that the AKH/AKHR and ACP/ACPR signaling systems are more closely related to one another^17,25^. To date, no functions have been assigned to the ACP signaling system, but expression profiles of ACP and ACPR in *R. prolixus*, and *T. castaneum*, show that both peptide and receptor are primarily expressed in the nervous system and, to a lesser extent, in the reproductive tissue of *R. prolixus*^17,25^. Furthermore, ACP transcript was detected in the head and thorax of adult *Aedes aegypti* with a similar transcript distribution observed in 4^th^ instar larvae, pupae, and adult *A. gambiae*^20,21^. Developmental expression profiles have revealed high expression of both ACP and its receptor before and after hatching of eggs in *T. castaneum* and after ecdysis in *R. prolixus*, and thus a role in early larval development and post-ecdysis, respectively, has been proposed^17,25^. Additionally, assays testing the potential for cardio-excitatory or lipid mobilization roles of ACP in *R. prolixus* yielded negative results, which indicates that ACP, AKH, and CRZ are indeed independent signaling systems with distinct functions^10^. Further investigations are clearly necessary in order to assign a physiological role for the ACP/ACPR neuropeptide system in insects.

*Aedes aegypti* mosquitoes are principal vectors for a variety of pathogens including dengue fever, yellow fever, chikungunya, and Zika viruses, all of which have a significant impact on human morbidity and mortality^26^. A thorough understanding of mosquito biology is required to devise novel methods to reduce and prevent mosquito-borne diseases. In an attempt to advance our understanding of the ACP signaling system in *A. aegypti*, we have identified and functionally deorphanized the *A. aegypti* ACPR (*Aedae*ACPR). Furthermore, to begin identification of prospective target tissues and physiological roles, we have determined the post-embryonic developmental expression profile and the spatial expression pattern in the adult mosquito of the transcripts encoding the ACP precursor peptide as well as a functional ACP receptor in *A. aegypti*.

## Materials and Methods

### Animals

*Aedes aegypti* (Liverpool strain) eggs were hatched in plastic containers half-filled with deionized water at an initial density of approximately 100 larvae/litre of water. Larvae were fed a 2% brewers yeast, 2% liver powder solution daily, and adults were provided with a 10% sucrose solution through a microcentrifuge tube fitted with a cotton ball wick allowing feeding *ad libitum*. Larvae and pupae were maintained in an incubator at 26 °C on a 12:12 hour light: dark cycle. Colony upkeep involved adult females being fed sheep’s blood in Alsever’s solution weekly (Cedarlane Laboratories Ltd., Burlington, ON) using an artificial feeding system described previously^27^. All experiments on adults were performed on either one or four-day old male and female mosquitoes that were sucrose-fed only and had been isolated during the pupal stage and transferred *en masse* into small glass microchambers.

### Isolation and cloning of cDNA encoding A. aegypti ACPR

Gene specific forward and reverse primers were designed using Primer 3 in Geneious Software (Biomatters Ltd, Auckland, New Zealand) based on a predicted incomplete *A. aegypti* ACPR sequence (XM_001653870) described previously^17^ to amplify a 975 bp partial fragment using Q5 High Fidelity DNA Polymerase (New England Biolabs, Whitby, On) and whole adult female *A. aegypti* cDNA as template. The PCR product was purified, A-tailed, cloned into pGEM-T vector (Promega, Madison, WI, USA) and nucleotide sequence was confirmed by Sanger sequencing (Center for Applied Genomics, Hospital for Sick Children, Toronto, ON). After successful validation of the cloned partial sequence, primers were designed (as mentioned above) to perform 5′ and 3′ rapid amplification of cDNA ends (RACE)-PCR utilizing the Clontech SMARTer 5′/3′ RACE Kit (Takara BIO USA Inc, CA, USA). To facilitate cloning of amplicons, the linker sequence GATTACGCCAAGCTT, which overlaps with the pRACE vector provided in the kit, was added to the 5′ ends of the gene specific primers (Table 1). First-strand cDNA synthesis was prepared using 1μg total RNA from adult female head using the 3′ CDS primer (provided in the kit) and a gene-specific reverse primer to generate template cDNA for 3′ and 5′ RACE, respectively. Initial attempts at 5′RACE using the 5′CDS primer (provided in the RACE kit) for first-strand cDNA synthesis and subsequent PCR with the SeqAmp DNA Polymerase (Takara BIO USA Inc, CA, USA) was not successful for this target. Thus, the protocol was modified to generate first-strand cDNA using a gene-specific reverse primer (*Aedae*ACPR-R1, Table 1) and subsequent nested RACE-PCR reactions utilized Q5 High Fidelity DNA Polymerase *in lieu* of SeqAmp DNA Polymerase. Nested PCR reactions utilized gene specific forward (3′ RACE) and reverse (5′ RACE) primers and a universal primer mix (UPM) to amplify the complete cDNA encoding *A. aegypti* ACPR. Optimal PCR cycling parameters for subsequent amplification of ACPR were determined empirically. Specifically, for 3′ RACE this included an initial denaturation at 94 °C for 1 min, followed by 40 cycles of 30 s at 94 °C, 30 s at 68 °C, and 3 min at 72 °C to amplify PCR products using SeqAmp DNA Polymerase. For 5′ RACE, the Q5 High Fidelity DNA Polymerase was utilized with the following cycling parameters, 30 s at 98 °C, followed by 30 cycles of 5 s at 98 °C, 15 s at 65–68 °C, 1 min 10 s at 72 °C, with a final extension step of 2 min at 72 °C. Following two rounds of PCR using nested gene-specific primers, amplicons were gel extracted and cloned into the linearized pRACE vector and miniprep samples were then sent for sequencing. Finally, primers were designed at the 5′ and 3′ ends of the complete cDNA sequence (including UTRs) and at the start and stop codons of the sequence (region including only the open reading frame, excluding UTRs), and were used for subsequent PCR amplification of the receptor with Q5 High Fidelity DNA polymerase to confirm base pair accuracy.

**Table 1:** Primer information for oligonucleotides used for initial amplification of partial cDNA, 5′ and 3′ RACE and RT-qPCR analysis to determine temporal and spatial transcript expression.

Oligo Name	Sequence (5′- > 3′)	Function
AedaeACPR-F1^a^	GGTCACACCGAAACGACAGTGG	Amplification and cloning of partial AedaeACPR
AedaeACPR-R1^a^	TGGACCTCCTCTGGGCTGC	Amplification and cloning of partial AedaeACPR and 1st strand cDNA synthesis for 5′ RACE
AedaeACPR-R2	GACCGATTGGAGATTTCACAC	5′RACE
AedaeACPR-R3	CAGAACAGGTTGTAAGCCGTCT	5′RACE
AedaeACPR-R4	TCCACCGAGCATTATTTTGC	5′RACE
AedaeACPR-R5	TCCAGTGGGATCATGATGAAG	5′RACE
AedaeACPR-LR5	GATTACGCCAAGCTTCCTCCAGTGGGATCATGATGAAG	5′RACE and cloning of 5′ RACE amplicon
AedaeACPR-LF1	GATTACGCCAAGCTTGGTCACACCGAAACGACAGTGG	3′ RACE
AedaeACPR-LF2	GATTACGCCAAGCTTGTTGGATCGGTGCTTTGCTGTGAT	3′ RACE
AedaeACPR-LF3	GATTACGCCAAGCTT GGCTTACAACCTGTTCTGCGTGG	3′ RACE and cloning of 3′ RACE amplicon
AedaeACPR-ORFkozak-F	GCCACCATGTATCTTTTCGGCAGGATTGCG	ORF cloning for functional receptor assay
AedaeACPR-ORF-R	TGATTTATCATCGCCAGCCACC	ORF cloning for functional receptor assay
AedaeACPR-N4721-F	AGGAATGGCAGCACCG	5′-phosphorylated primers for generation of ACPR-I-N472I variant
AedaeACPR-N4721-R	GGCGAAGATCCCGTTG	5′-phosphorylated primers for generation of ACPR-I-N472I variant
AedaeACPR-qPCR-F	GGGATGCGACTTCGTTGTA	qPCR amplification of AedaeACPR-I
AedaeACPR-qPCR-R	TCGCGGTCAAACATGTACC	qPCR amplification of AedaeACPR-I
AedaeACP-qPCR-F^b^	ATGTGTTCTCTAAGGCGAAATAGC	qPCR amplification of AedaeACP
AedaeACP-qPCR-R^b^	TTACAGGTGCCCATTCGAA	qPCR amplification of AedaeACP

^a^Primers based on partial ACPR mRNA sequence (Accession number: XM_001653870.2^16^) identified through homology based *in silico* analysis of the *A. aegypti* genome.

^b^Primers based on ACP mRNA sequence (Accession number: FM391984.1^19^).

### Gene Structure and Phylogenetic Analyses

Mapping of exon-intron boundaries of *A. aegypti ACPR* gene was determined using the cloned complete cDNA sequence as a query against the *A. aegypti* genome scaffolds database available locally on a lab computer running Geneious Pro Bioinformatics Software (Biomatters Ltd, Auckland, New Zealand). Positions of introns and exons were further confirmed using the BDGP splice site prediction server using the standardized data set of *D. melanogaster* genes^28^. Membrane topology of ACPR-I, II, and III were predicted using the Constrained Consensus TOPology prediction server (CCTOP)^29^. The deduced *Aedae*ACPR-I, II and III protein sequences were aligned to the human gonadotropin-releasing hormone receptor 1 along with ACP, AKH, and CRZR receptors from other species (see Table S1) using ClustalW in MEGA 6.06^30^. Relationships between the various receptor sequences were determined through neighbour-joining^31^ and maximum-likelihood phylogenetic analysis methods^32^. Bootstrap values are based on 1000 replicates.

### Preparation of mammalian expression constructs

Amplicons encoding just the open reading frame (start ATG to stop codon) were used as template for re-amplification using a modified forward primer possessing the consensus Kozak translation initiation sequence^33,34^ at the 5′ end of the start codon. The resulting product was cloned into pGEM-T Easy vector and then subcloned into the mammalian expression vector, pcDNA 3.1^+^ (Life Technologies, Burlington, ON). Construct directionality was confirmed by Sanger sequencing and plasmid DNA was purified from overnight bacterial cultures using a PureLink MidiPrep Kit (Invitrogen, Burlington, ON) and subsequently used for transfection of mammalian cells for the receptor functional assay. Analysis of multiple independent sequences obtained from RACE-PCR revealed a number of single nucleotide polymorphisms (SNPs) that localized to various sites along the full cDNA sequence. Upon further analysis, it was determined that only a single SNP at nucleotide position 1924 within the open reading frame resulted in an amino acid change. To determine whether this difference in the resulting residue, in comparison to the *A. aegypti* genome database, confers any difference to the functional activity of the receptor, site directed mutagenesis was performed. Specifically, 5′ phosphorylated primers were designed (Table 1) with the forward primer possessing an adenine (position 1924) consistent with the *A. aegypti* genome sequence whereas our consensus sequence contained a thymine in this nucleotide position. Using these modified primers, asymmetric PCR was performed using a pGEM-T Easy plasmid construct as template to replace the Ile_472_ in the cloned receptor with an Asn_472_ matching the *A. aegypti* genome. Mutation of the coding sequence was verified by sequencing and sub-cloned into the mammalian expression plasmid, pcDNA3.1^+^ (as described above).

### Cell culture, transfections, and bioluminescence assay

Functional activation of *Aedae*ACPR-I was assayed using a previously established cell culture system involving a recombinant Chinese hamster ovary (CHO)-K1 cell line stably expressing aequorin^35^. Cells were maintained in Dulbecco’s modified eagles medium: nutrient F12 (DMEM:F12; 1:1) media containing 200ug/mL geneticin, 10% heat-inactivated fetal bovine serum (FBS; Wisent, St. Bruno, QC) and grown to approximately 90% confluency, and were transiently transfected with pcDNA3.1^+^ expression vector possessing *Aedae*ACPR-I using Lipofectamine LTX and Plus Reagent transfection system (Invitrogen, Burlington, ON) following a 3:1:1 transfection reagent (μL): Plus reagent (μL): plasmid DNA (μg) ratio. At 48 hours post-transfection, cells were detached from the culture flasks using Dulbecco’s phosphate buffered saline (DPBS; Wisent Corporation, St. Bruno, QC) containing 5 mM EDTA and resuspended in BSA medium (DMEM-F12 media containing 0.1% bovine serum albumin, 1X antimycotic-antibiotic) to a concentration of 10^6^–10^7^ cells/mL. Coelenterazine h (Promega, Madison, WI, USA) was added to the cells to a final concentration of 5 μM, and incubated for 3 hours in the dark at room temperature on a stirrer set at 200 rpm. The cell suspension was then diluted 10-fold and incubated at room temperature for an additional hour. Serial dilutions of peptide ligands (Table 1) were prepared in BSA medium (10^−5^ to 10^−12^ M), and loaded in quadruplicates into 96-well luminescence plates (Greiner Bio-One, Germany). All peptides were commercially synthesized (Genscript, Piscataway, NJ) at a purity >90% and were prepared in dimethyl sulfoxide at a stock concentration of 1 mM. Cells were loaded into each well with an automatic injector unit and luminescence was measured for 20 seconds using a Synergy 2 Multi-Mode Microplate Reader (BioTek, Winooski, VT, USA). BSA medium alone was utilized as a negative control and 5 × 10^−5^ M ATP was used as a positive control, which acts on endogenously expressed purinoceptors^36,37^. EC_50_ values were calculated in GraphPad Prism 7.02 (GraphPad Software, San Diego, USA) from dose-dependent curves from four independent transfections.

### Tissue dissections, RNA extraction, and cDNA synthesis

Lightly CO_2_-immobilized four-day old adult male (n = 30) and female (n = 20) *A. aegypti* were submerged in DPBS, and the following body segments and/or tissues were dissected and isolated: head, midgut, Malpighian tubules, hindgut, ovaries, testes, accessory reproductive tissues, and carcass (remaining fat body, musculature, and cuticle). Tissues were lysed in RNA lysis buffer containing 1% 2-mercaptoethanol. Whole adult RNA was obtained from submerging several males and females in RNA lysis buffer containing 1% 2-mercaptoethanol and using a sterile plastic pestle to disrupt the tissue. To measure the developmental expression profile for *Aedae*ACPR, first to fourth instar larvae, pupae, as well as one- and four-day old adult mosquitoes were collected and submerged in RNA lysis buffer and flash frozen in liquid nitrogen. Total RNA was isolated from whole animal and individual adult tissues samples mentioned above using the PureLink^TM^ RNA mini kit following manufacturer protocol with an on-column DNase treatment to remove genomic DNA (Invitrogen, Burlington, ON). Purified total RNA samples were quantified with a Take3 micro-volume plate and measured on a Synergy Multi-Mode Microplate Reader (BioTek, Winooski, VT, USA). To assess *ACP* and *ACPR* transcript levels, cDNA was synthesized from 20 ng total RNA using the iScript™ Reverse Transcription Supermix for RT-qPCR (Bio-Rad, Mississauga, ON) following manufacturers protocol, including a ten-fold dilution of cDNA following synthesis.

### Quantitative PCR

ACP and ACPR transcript abundance was quantified on a StepOnePlus™ Real Time PCR system (Applied Biosystems, Carlsband, CA) using PowerUP™ SYBR® Green Master Mix (Applied Biosystems, Carlsband, CA). Cycling conditions were as follows: 1) UDG activation 50 °C for 2 min, 2) 95 °C for 2 min, and 3) 40 cycles of (i) 95 °C for 15 seconds and (ii) 60 °C for 1 minute. Gene-specific primers designed over different exons were used to amplify *ACPR*, with the forward primer designed over exon 5 (nucleotides 1458–1476) to ensure specificity for ACPR-I, and the reverse primer over exon 6 (nucleotides 1585–1603). Gene specific primers amplifying *Aedae*ACP were designed over multiple exons (Table 1; forward: nucleotides 89–112, reverse: nucleotides 403–421) based on a previously published mRNA sequence (Genbank Accession Number: FN391984)^20^. Relative expression levels were determined using the ΔΔC_T_ method and were normalized to the geometric mean of *rp49*, *rpL8*, and *rps18* reference genes, which were previously characterized and determined as optimal endogenous controls^38^. The *AedaeACPR* spatial expression profile was determined using 7–9 biological replicates, all of which included three technical replicates per reaction and a no-template negative control. The *AedaeACPR* developmental expression is an average of 3–5 biological replicates that each included duplicate technical replicates for each target gene and a no-template negative control. The *AedaeACP* spatial expression profile consisted of 3–4 biological replicates and the developmental expression is an average of 3–5 biological replicates. Specificity of primers for target mRNA were assessed by conducting no reverse-transcriptase controls, analysis of dissociation curves, and Sanger sequencing of amplicons. Data were analyzed using a one-way ANOVA with Dunnett’s multiple comparisons test where p < 0.05 was considered significant.

### Data availability

The datasets generated and analysed during the current study are available from the corresponding author on reasonable request.

## Results and Discussion

**Figure 1:**
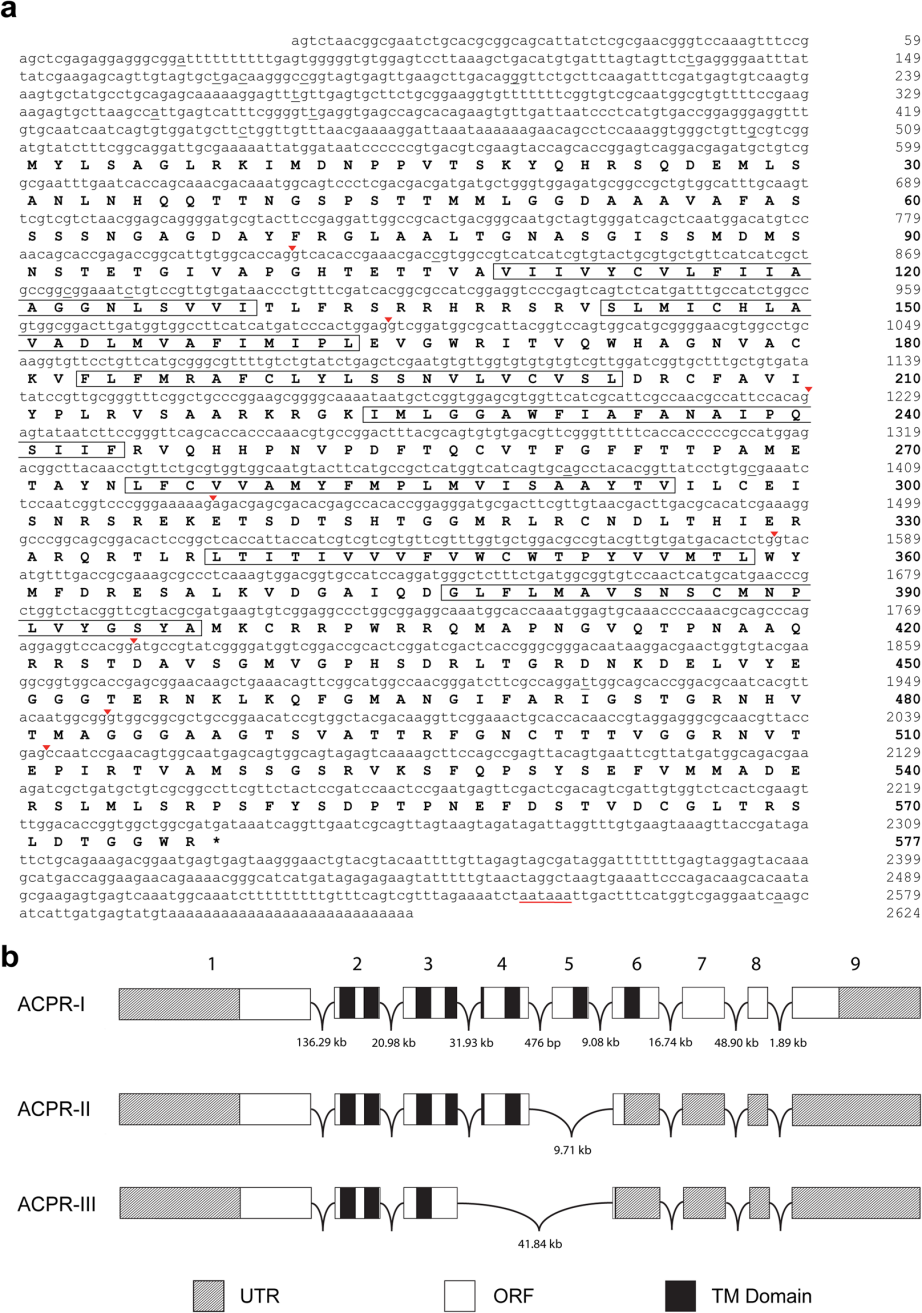
*Aedes aegypti* adipokinetic hormone/corazonin-related peptide receptor variant I (ACPR-I) cDNA, amino acid sequence, and predicted gene structure. (**a**) Lower case letters denote cDNA, and uppercase letters represent amino acid residues with positions denoted by the numbers on the right of the sequences with bolded numbers indicating amino acid positions. Exon-exon boundaries within the cDNA are denoted by inverted red arrowheads. Underlined nucleotides indicate single nucleotide polymorphisms that differed from the *A. aegypti* genome. A putative polyadenylation signal is underlined in red in the 3′UTR. The predicted hydrophobic alpha-helices that form the transmembrane domains are outlined by black rectangular boxes. (**b**) The splicing pattern of the *Aedae*ACPR gene based on BLAST analyses of cloned cDNA and splice site prediction analysis. Alternative splicing gives rise to three receptor mRNA variants where *ACPR-I* possesses all exons, *ACPR-II* lacks exon 5, and *ACPR-III* lacks both exons 4 and 5. Boxes representing exons are drawn to scale whereas intervening introns are not drawn to scale.

We have identified the complete cDNA sequence encoding the *A. aegypti* adipokinetic hormone/corazonin-related peptide (ACP) receptor (Fig. 1a). Following initial cloning and sequence analysis, three transcript variants were identified, *Aedae*ACPR-I, ACPR-II, and ACPR-III (Fig. 1b). *Aedae*ACPR-I is 2596 bp (GenBank Accession number: MF461644), which includes a 1734 bp open reading frame (ORF) encoding a 577 residue receptor (Fig. 1a). The cloned 5′ untranslated region (UTR) is 509 bp in length and the 3′UTR is 346 bp and contains a predicted polyadenylation sequence (nucleotide position 2547–2552). *Aedae*ACPRs II and III are transcript variants of 2442 bp (GenBank: MF461645) and 2240 bp (GenBank: MF461646) in length, which yield deduced proteins comprised of 328 and 243 amino acids, respectively. Only *Aedae*ACPR-I has the seven expected hydrophobic transmembrane (TM) domains characteristic of GPCRs, whereas *Aedae*ACPR-II has only five TM domains and *Aedae*ACPR-III has only three TM domains (see Fig. S1). Similarly, previous studies in *R. prolixus*^25^ and *A. gambiae*^17^ revealed multiple receptor variants, whereas only a single variant was identified in *T. castaneum*^17^. Specifically, in *R. prolixus*, three ACPR variants have been identified, two of which contain the typical seven TM domains (*Rhopr*ACPR-B and *Rhopr*ACPR-C), and one which contains five TM domains^25^. Additionally, two ACPR isoforms have been identified in *A. gambiae*, *Anoga*ACPR-A and *Anoga*ACPR-B, both of which result in receptors possessing seven TM domains^17^.

The gene structure was modeled by comparing the cloned cDNA sequence to the *A. aegypti* genome database using Geneious Bioinformatics software (Biomatters Ltd., Auckland, New Zealand). Analysis of the *A. aegypti* ACPR-I receptor shows that it maps over nine exons that are 810 bp, 191 bp, 228 bp, 202 bp, 154 bp, 197 bp, 177 bp, 83 bp, and 549 bp long, respectively. Alternative splicing of the *A. aegypti* ACPR gene results in the absence of either exon 5 only or absence of both exons 4 and 5, which yields *Aedae*ACPR-II and *Aedae*ACPR-III, respectively, both resulting in-frame translation shifts and premature stop codons, and consequently, truncated ORFs (Fig. 1b).

**Figure 2:**
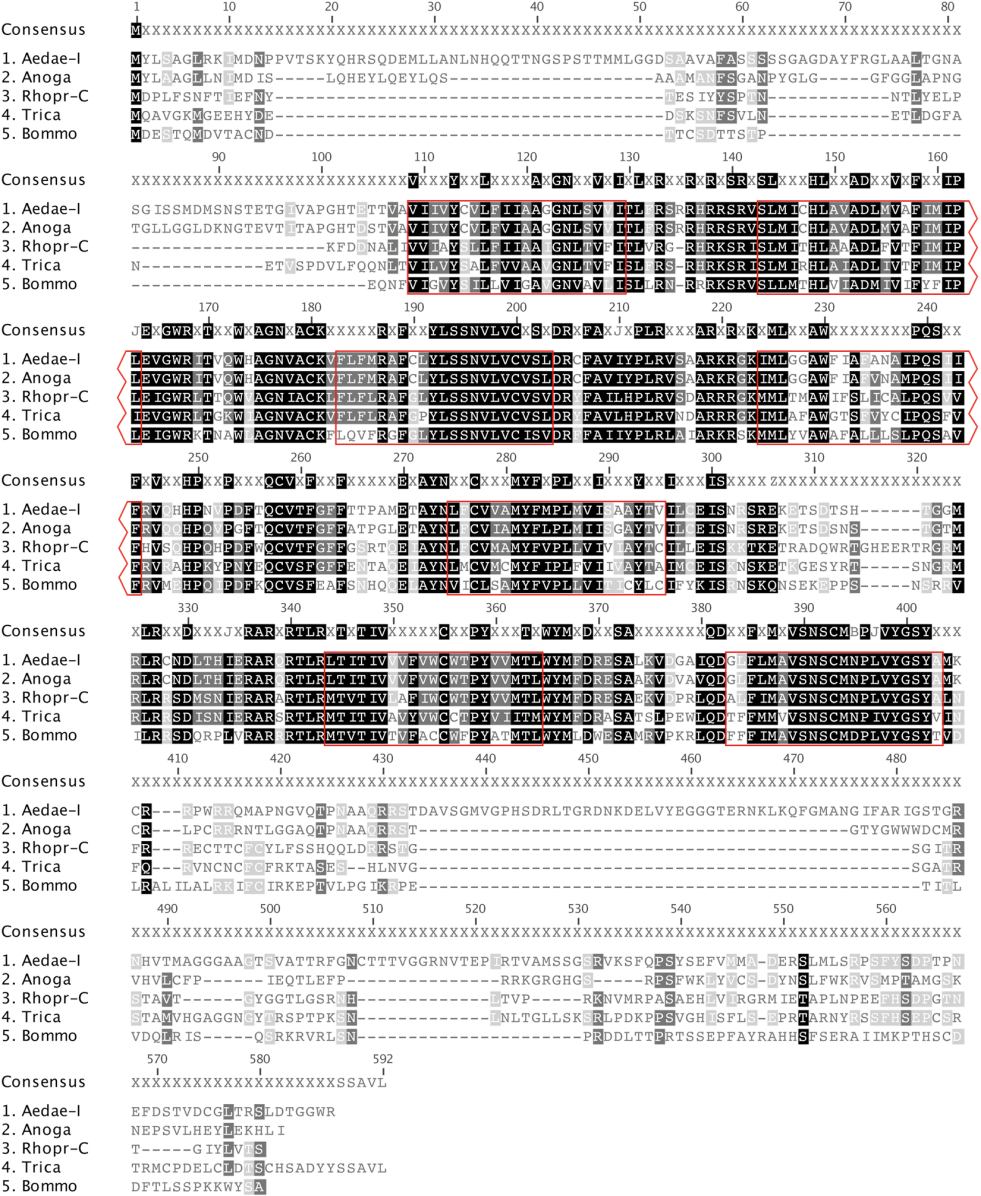
Sequence alignment of select insect adipokinetic hormone/corazonin-related peptide receptors (ACPR). Aligned amino acid sequences of ACPRs from *A. aegypti* (Variant I, GenBank: MF461644) *R. prolixus* (GenBank: AKO62858), *B. mori* (GenBank: NP_001127726), *T. castaneum* (GenBank: ABX52400), and *A. gambiae* (GenBank: ABX52399). Residues outlined in red indicate predicted transmembrane domains based on the *A. aegypti* ACPR sequence. Highlighting of residues indicates % identity with black denoting 100% sequence identity, dark grey denotes 80–100% identity, and light grey represents amino acid positions with 60–80% sequence identity.

Alignment of *A. aegypti* ACPR-I, with selected receptors from *A. gambiae*, *T. castaneum*, *R. prolixus*, and *B. mori*, reveals conservation of the ACP receptor across insect species (Fig. 2). Specifically, *Aedae*ACPR-I shares 59.4% sequence identity with the *A. gambiae* ACP receptor, 42.4% identity with the *R. prolixus* ACPR-C, 41.5% identity with the *T. castaneum* ACPR and 33.8% identity with the *B. mori* ACP receptor. Overall, there is a high degree of conservation over the seven predicted TM domains, particularly over TM regions one, two, three, five and seven. Strong sequence identity is also observed in the first and second intracellular loops, as well as the first extracellular loop. All of the receptor sequences, except for *Bommo*ACPR, which harbors an Asp in place of Asn, possess the conserved NPXXY motif in the seventh TM domain characteristic of rhodopsin-like (family A) GPCRs^39,40^. Another conserved motif found in rhodopsin-like GPCRs is the E/DRY motif adjacent to the second intracellular loop^40^. The Arg of the E/DRY motif and a negatively charged residue on TMVI of the GPCR undergo ionic interactions, known as the ionic lock, which stabilizes the inactive state of the receptor^41^. In particular, the ACP receptors possess a DRF motif in the silkworm *B. mori*, and DRC motifs are found in the mosquitoes *A. gambiae* and *A. aegypti*, in place of the characteristic DRY motif found in hemipteran *R. prolixus* and coleopteran *T. castaneum*, which have more conserved features of rhodopsin-like GPCRs^40,42^.

**Figure 3:**
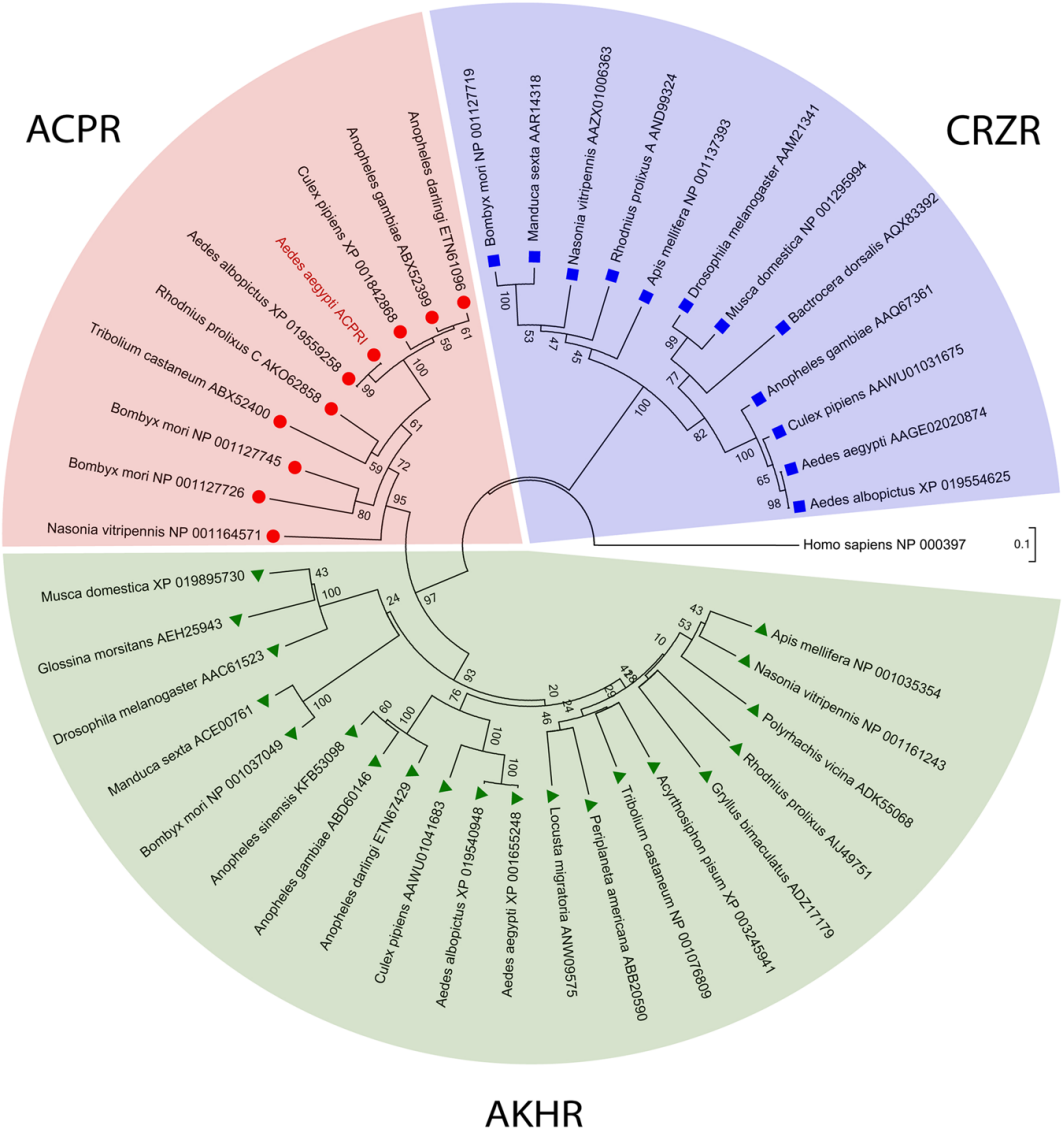
Phylogenetic relationship of adipokinetic hormone receptors (AKHR, ), corazonin receptors (CRZR, ) and adipokinetic hormone/corazonin-related peptide receptors (ACPR, ) from insects. Tree was constructed using the neighbour-joining analysis (with 1000 bootstrap replicates). Branch lengths indicate the number of amino acid substitutions per site and numbers adjacent to nodes denotes the percentage support for the clustering of the related sequences in that particular clade. Receptor protein sequences are labelled by species name and identified with their GenBank accession number. The *Aedae*ACPR-I receptor cloned in this study is denoted in red text. The human gonadotropin releasing hormone receptor isoform 1 (GenBank: NP_000397) was included in the analysis and designated as the outgroup.

Phylogenetic analysis using the neighbor-joining and maximum-likelihood methods (not shown) yielded trees with highly similar and well supported topologies (Fig. 3). All the ACP receptors analyzed are positioned within a single clade that is a sister group to the clade comprised of the AKH receptors. Together, the AKH and ACP receptor clades form a monophyletic group which is a sister group to the clade comprised of CRZ receptors. The *Aedae*ACPR-I identified herein clusters closely with the other insect ACPRs that have previously been identified and functionally characterized confirming that the receptor isolated in this study is an ortholog of other insect ACP receptors^17,18,22,25^. Predicted ACPRs from other mosquito species including, *Anopheles darlingi*, *Culex pipiens*, and the Asian tiger mosquito *Aedes albopictus* also cluster closely to the *A. gambiae* and *A. aegypti* ACP receptors.

**Figure 4:**
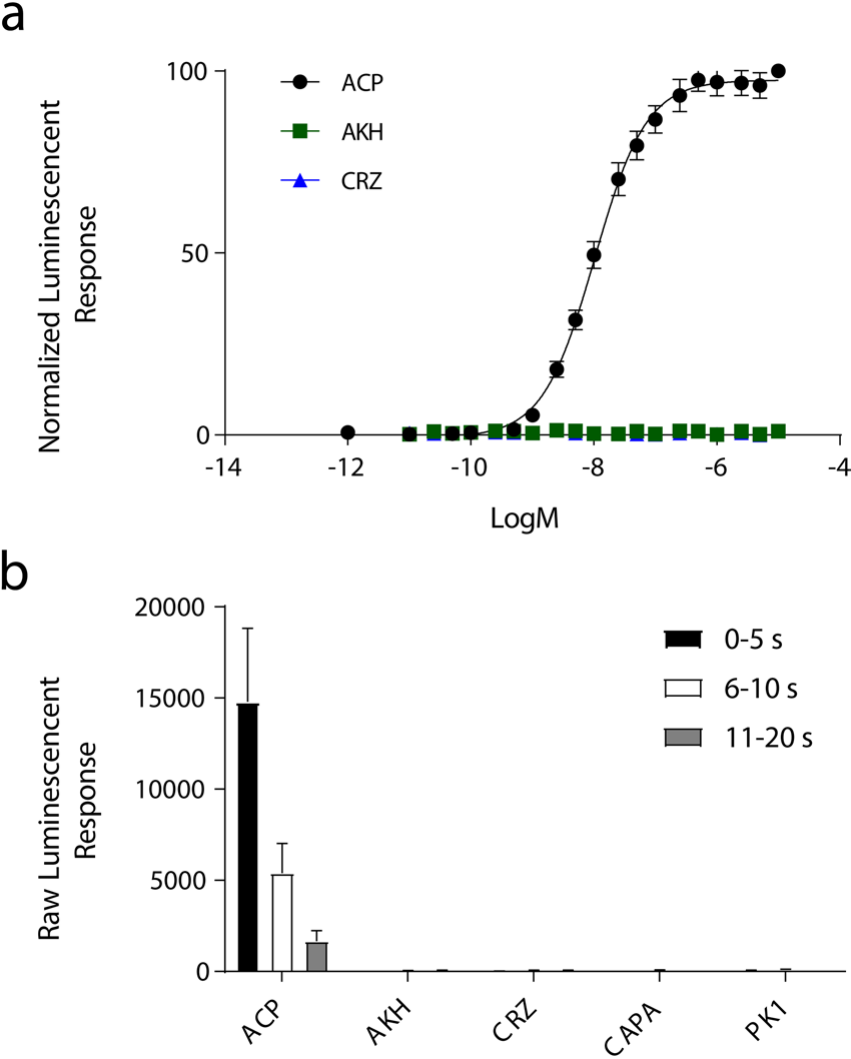
Functional heterologous receptor assay of CHO-K1 aequorin cells transiently expressing the *Aedae*ACPR-I. (**a**) Dose dependent effect on the bioluminescence response (mean 0–15 s) after the addition of between 10^−12^–10^−5^ M doses of *A. aegypti* ACP, AKH and CRZ peptides. Luminescence is normalized to the BSA control and plotted relative to the maximal response (10^−5^ M). The EC_50_ for *Aedae*ACP is 1.025 × 10^−8^ M. No receptor activation was detected when challenged with *Aedae*AKH and *Aedae*CRZ or *Aedae*CAPA1 and *Aedae*PK1 (not shown). (**b**) Kinetics of the bioluminescence response measured between 0–5 s, 6–10 s, and 11–20 s time intervals, following the addition of 1 × 10^−6^ M of the above peptides normalized to vehicle control (BSA media). Luminescence is normalized to the BSA control and plotted relative to the maximal response (10^−5^ M). Data represent the mean ± standard error (n = 4).

A heterologous receptor functional assay involving CHO-K1 cells was used to validate the cloned receptor as a bona fide ACP receptor. Indeed, the *Aedae*ACPR-I was dose-dependently activated by *Aedae*ACP (EC_50_ = 1.025 × 10^−8^ M) (Fig. 4a), confirming the proposed identity of the receptor based on phylogenetic analysis. Kinetic analysis of receptor activation demonstrated maximal luminescence response was evident over the first five seconds following application of the ACP peptide, indicative of an immediate and transient elevation of intracellular calcium levels elicited through activation of the ACP receptor (Fig. 4b). Our results also confirm the specificity of the ACP receptor for the ACP peptide alone (see Table 2), since no detectable luminescence indicative of receptor activation was observed in response to the closely related peptides, *Aedae*AKH and *Aedae*CRZ, or other tested peptides, specifically *Aedae*CAPA-1 and pyrokinin-1 (*Aedae*PK1), which share no structural similarity to *Aedae*ACP. Our findings in this study are consistent with past reports, since similar binding specificity of ACP receptors has been observed previously in *T. castaneum*, *A. gambiae*, and *R. prolixus* with EC_50_ values reported in the low nanomolar range^17,25^. Additionally, previous research in the aforementioned insects have also observed that the AKH receptors are not activated by ACP or corazonin peptides, and similarly, the corazonin receptors are not activated by ACP or AKH peptides^17,25,43,44^. Thus, consistent with these previous observations, we determined that although these neuropeptide systems are structurally and evolutionarily related, they are indeed independent of one another and do not exhibit any cross talk in *A. aegpyti*. Notably, however, previous studies in *B. mori* have revealed that high concentrations of *Bommo-*ACP (previously referred to as AKH3) resulted in the activation of *Bommo-*AKHR whereas sensitivity to its natural AKH ligand was approximately 100-fold higher^23^. Similarly, high doses of the AKH peptides in *B. mori*, *Bommo*-AKH1 and *Bommo*-AKH2, were also found to activate putative *B. mori* ACPRs (A28 and A29), albeit at significantly higher concentrations than ACP^22^.

**Table 2:** Structure of peptides tested in the heterologous receptor functional assay and summary of activity in eliciting a luminescent response.

Peptide Name	Peptide Sequence	EC_50_ (nM)^a^
*Aedae*ACP	pQVTFSRDWNAa	10.25
*Aedae*AKH	pQLTFTPSWa	NA^b^
*Aedae*CRZ	pQTFQYSRGWNTNa	NA^b^
*Aedae*CAPA1	GPTVGLFAFPRVa	NA^b^
*Aedae*CAPA-PK1	AGNSGANSGMWFGPRLa	NA^b^

^a^EC_50_ values are the averages of multiple independent biological replicates involving CHOK1-aeq cells transiently expressing *Aedae*ACPR-I.

^b^No activity (NA) detected when tested with peptide titres up to a maximum of 10 µM.

In comparison to the *A. aegypti* genome, a number of single nucleotide polymorphisms (SNP) were observed across the entire cDNA sequence. Of those occurring within the open reading frame, only one SNP (nucleotide 1924, found within the seventh exon, which corresponds to the C-terminus of the receptor) results in a different amino acid at residue Ile_472_, compared to the Asn_472_ predicted by the *A. aegypti* genome. Modification of the isoleucine residue (Ile_472_) obtained in our cDNA to the genome consistent asparagine residue (Asn_472_) resulted in no change to receptor activation by its endogenous ACP ligand, as determined by equal luminescent response by both the cloned *Aedae*ACPR-I and the mutated *Aedae*ACPR-I-N472I (Fig. S2). No luminescence signals were obtained in response to any of the tested peptides in untransfected cells, or cells transfected with empty pcDNA 3.1^+^ vector (data not shown).

**Figure 5:**
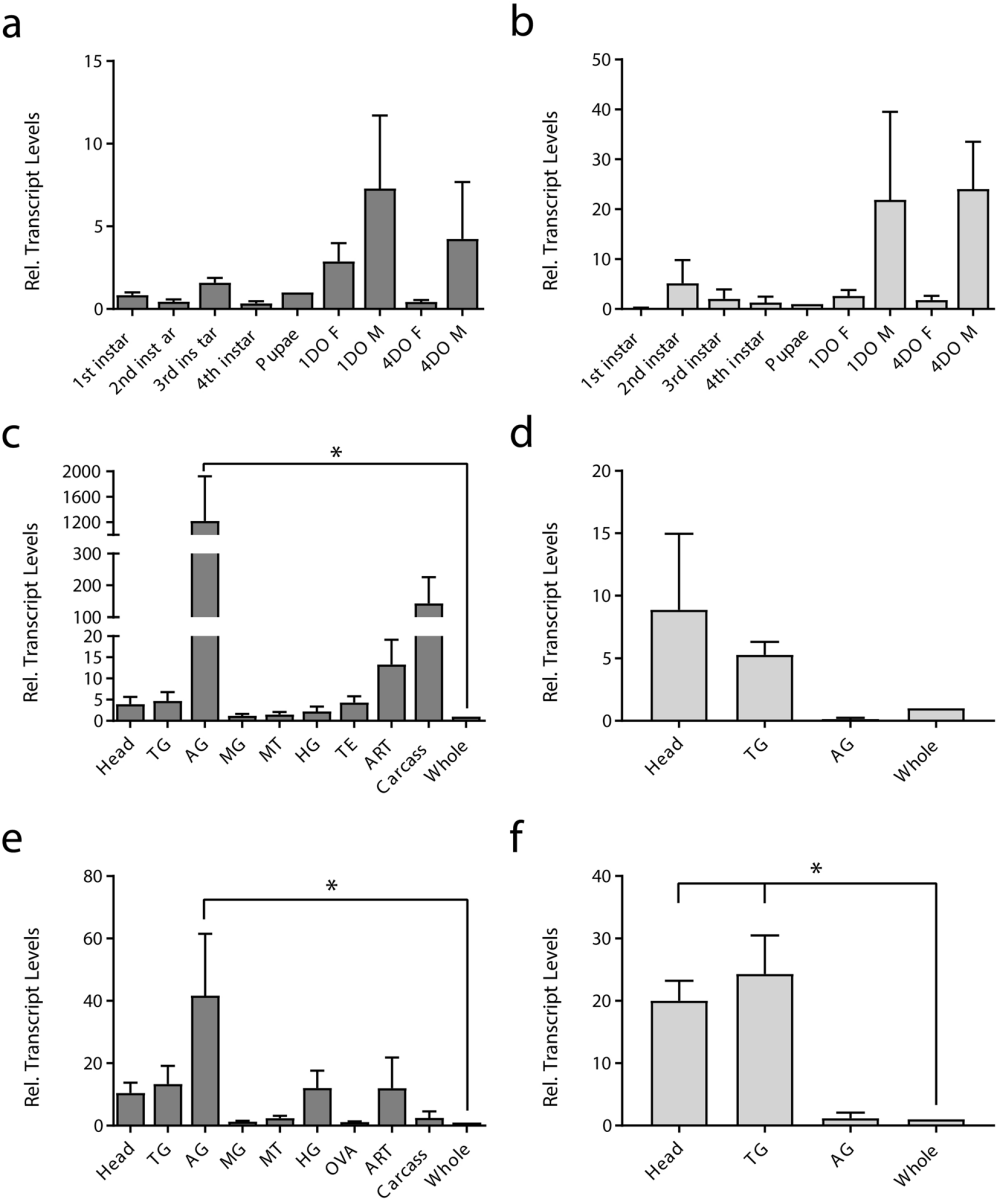
Transcript expression pattern of *ACPR-I* and *ACP* during post-embryonic development and in specific tissues of four-day old adult *A. aegypti*. Temporal expression of *ACPR-I* (**a**) and *ACP* (**b**) transcript is analyzed across developmental stages of the mosquito with expression shown relative to transcript levels in pupa. Spatial expression is analyzed in various tissues from four-day old adult males, *ACPR-I* (**c**) and *ACP* (**d**), and females, *ACPR-I* (**e**) and *ACP* (**f**) with transcript abundance shown relative to levels in whole body adult mosquitoes. Abbreviations: thoracic ganglia (TG), abdominal ganglia (AG), midgut (MG), Malpighian tubules (MT), hindgut (HG), ovaries (OVA), testes (TE), accessory reproductive tissue (ART). Data represent mean ± standard error, *denotes significance (p < 0.05) as determined by a one-way ANOVA, Dunnett’s multiple comparisons test.

Next, utilizing RT-qPCR, we investigated the molecular expression of the ACP signaling system during development and in individual tissues of adult *A. aegypti*. Although we identified three transcript variants, only the *ACPR-I* transcript yields a complete receptor protein which we functionally deorphanized, and so expression profiles were determined only for this transcript variant (see methods). Developmental expression profiling revealed enrichment of both *ACPR-I* (Fig. 5a) and *ACP* (Fig. 5b) transcripts following the transition from pupal to adult stages. In particular, one-day and four-day old male *A. aegypti* had the highest levels of *ACP* and *ACPR-I* transcript abundance. Similar findings for the ACP receptor were observed in *R. prolixus*^17,25^; however, in contrast, *ACPR* transcript levels in *T. castaenum* were highest in late embryonic and early larval stages and decreased thereafter as the beetle progressed in development^17,25^. In *A. aegypti*, the observed enrichment of the *ACP* and *ACPR-I* transcripts could be indicative of a post-eclosion function for the ACP system.

Spatial transcript expression profiles of *A. aegypti ACPR* aimed to reveal potential functional roles for ACP and its receptor by determining prospective target tissues. The ACP receptor (*ACPR-I*) was found to be significantly enriched in the abdominal ganglia of both male (Fig. 5c) and female (Fig. 5e) *A. aegypti* when compared relative to expression in the whole adult (males, p = 0.0025 and for females, p = 0.0016). *ACPR-I* transcript was also observed in the carcass, accessory reproductive tissue, testes, head and thoracic ganglia of adult male mosquitoes, although not significantly enriched as was found in the abdominal ganglia (Fig. 5c). Similarly, *ACPR-I* transcript was also found in the head, thoracic ganglia, hindgut, and accessory reproductive tissues of adult female *A. aegypti* (Fig. 5e). Enrichment of the ACP receptor transcript in the nervous system is not surprising, since expression of the transcripts encoding the ACP receptor and peptide have been described in the nervous system of other insects. Specifically, in fifth instar and adult *R. prolixus*, ACPR transcript was found to be enriched in the central nervous system and the corpus cardiacum /corpora allata (CC/CA) complex^25^. *ACPR* expression in *T. castaneum* was revealed to be greatest in the brain in comparison to the torso (i.e. body minus the head)^17^. *AedaeACP* transcript was detected in the central nervous system, where it was enriched in the brain and thoracic ganglia of male (Fig. 5d) and significantly enriched in the head (p = 0.0127) and thoracic ganglia (p = 0.004) of female mosquitoes (Fig. 5f). Consistent with our quantified ACP tissue-specific expression profile, ACP transcripts in *A. aegypti* and *A. gambiae* were detected solely in head and thorax body segments of adult mosquitoes^20,21^.

Expression of *A. aegypti ACPR-I* within the central nervous system suggests that ACP may be functioning as a neuromodulator and/or a neurotransmitter. This possibility is supported by the extensive varicose immunoreactive staining of ACP in the central nervous system of *T. castaneum* first instar larvae, where a neurosecretory role was suggested^17^. Specifically, immunoreactivity (IR) was observed in three to four neurons in the brain, the central brain neuropil with projections from the brain descending into the suboesophageal ganglion (SOG), thoracic ganglia, and abdominal ganglia, with no projections observed exiting the nervous system^17^. Immunocytochemistry using an antiserum against *D. melanogaster* AKH, which recognizes both AKH and ACP in *A. gambiae* and *A. aegypti*^20,21^, revealed immunoreactivity throughout the mosquito nervous system. In both *A. aegypti* and *A. gambiae*, AKH-like immunoreactivity was observed in two pairs of lateral neurosecretory cells in the brain, but was explained by the authors of this study to represent ACP-producing neurons, since AKH synthesis and storage is restricted to the corpus cardiacum^20,21^. Within the fused thoracic ganglia of adult *A. aegypti* and *A. gambiae*, AKH-like immunoreactivity was observed within at least one to a few cells within the ventral region of the pro- and mesothoracic ganglia^20,21^. Thoracic extracts were negative for ACP-like activity in *A. gambiae*^20,21^, however, expression of the AKH transcript in adult male *A. aegypti* is absent in the head and thorax region^20,21^; thus, it is unclear if the cells detected previously within the thoracic ganglia are ACP- or AKH-producing neurons in mosquitoes although our data indicate that the *A. aegypti* ACP transcript is prominently expressed in both the brain and thoracic regions of the nervous system. In *R. prolixus*, ACP-like immunoreactivity is observed in two bilaterally paired cell bodies at the anterior portion of the protocerebrum near the optic lobes, and one bilateral pair of cell bodies medially positioned in the protocerebrum^10^. Considering the extensive immunohistochemical distribution of ACP throughout the nervous system of insects^16,19,20^, the prominent expression of *ACP* transcript in the brain and thoracic ganglia along with the significant enrichment of *ACPR-I* within the abdominal ganglia of adult *A. aegypti* strongly supports that ACP may be functioning centrally within the nervous system. In *L. migratoria* ACP was identified in the storage lobe of the CC, in contrast to the glandular lobe where AKH is found, suggesting synthesis of this neuropeptide within neurosecretory cells of the brain^18^. Furthermore, it was previously suggested that given *ACPR* was found in the CC/CA complex in *R. prolixus*, ACP may be involved in the regulation of other hormones in a manner similar to its distant vertebrate homolog, GnRH^25^.

We also determined *A. aegypti ACPR-I* expression is not restricted to nervous tissue since transcript expression was detected in other tissues/organs including the female hindgut (Fig. 5e) and male carcass (Fig. 5c). ACPR expression in the hindgut, the primary site of reabsorption of ions and metabolites^45^, was unexpected since neither AKH nor CRZ have been found to regulate hydromineral balance. Thus, this possible function for ACP on the hindgut will require further investigation. Detection of the ACPR transcript in the carcass, which includes the body wall musculature and fat body, suggests that ACP and AKH may share a lipid mobilizing function. However, this possibility is unlikely since ACP was shown to have no influence on lipid or carbohydrate metabolism in female *A. gambiae* nor did it influence energy stores in male insects of *L. migratoria* or *P. americana*^46^. Interestingly, contrary to our predictions, both spatial and temporal expression profiles reveal greater expression of *ACP* and *ACPR* in adult males compared to females, which is consistent with an earlier microarray analysis in *A. gambiae* that determined higher ACP expression in adult males, compared to adult female and last instar larvae^47^. There is no clear explanation for such a sex-specific difference in *ACP* and *ACPR* transcript expression, however similar to our findings, male *D. melanogaster* express greater levels of the AKH receptor than their female counterparts^8^. *ACPR-I* transcript expression was also observed in the accessory reproductive tissues of both male and female *A. aegypti*. Expression of ACPR in reproductive tissue has also been observed in *R. prolixus*^25^. Perhaps, in addition to structural homology between ACPR and GnRHR, there is a yet undiscovered functional conservation between these two signaling systems. Furthermore, in *Gryllus bimaculatus*, pharmacologically elevating AKH titre through injections resulted in a significantly lower egg production^48^. In *Caenorhabditis elegans*, knockdown of the AKH-GnRH peptide and GnRH receptor resulted in reduced progeny in early worms^49^. Also, in *Glossina morsitans*, knockdown of the AKHR transcript resulted in higher levels of whole body lipids and, in pregnant flies, the inability to utilize lipid reserves resulted in delayed larval development and thus reproductive disruption^50^. Whether these effects on reproduction in these organisms are a direct result of signalling involving the AKH or GnRH receptor or AKH peptide remains unclear. Additionally, analysis of seminal fluid protein content of *A. albopictus* revealed the AKH peptide as one of the proteins transferred from males to female mosquitoes during mating^51^. Recently, CRZR transcript expression in *R. prolixus* was also observed in male and female reproductive tissues, which suggests some potentially overlapping reproductive target tissues in insects^44^.

Currently, no definitive function for ACP has been determined and functional studies in other insects have revealed that ACP does not influence energy mobilization and so does not duplicate the actions of AKH^10,46^. Additionally, ACP failed to increase heart-beat frequency, suggesting that the physiological actions of ACP does not mirror the most established function of CRZ^10^. Further studies are necessary to unravel the function of the ACP system, which could include methods aimed at knockdown of the ACP peptide or receptor.

## Electronic supplementary material

Supplementary information
