# Supplementary material for: Molecular identification, transcript expression, and functional deorphanization of the adipokinetic hormone/corazonin-related peptide receptor in the disease vector, Aedes aegypti
Source: Sci Rep. 2018 Feb 1;8:2146. doi: 10.1038/s41598-018-20517-8 (PMC5794978; doi:10.1038/s41598-018-20517-8)
Supplement: Supplementary file 1 — Supplementary information [file 41598_2018_20517_MOESM1_ESM.pdf]

Molecular identification, transcript expression, and functional deorphanization of the adipokinetic hormone/corazonin-related peptide receptor in the disease vector, *Aedes aegypti*.

Azizia Wahedi and Jean-Paul Paluzzi

SUPPLEMENTARY INFORMATION

```
           1         10        20        30        40        50        60
Consensus  MYLSAGLRKIMDNPPVTSKYQHRSQDEMLLANLNHQQTTNGSPSTTMMLGGDSAAVAFASSSSSGAGDA

1. Aedae-I    MYLSAGLRKIMDNPPVTSKYQHRSQDEMLLANLNHQQTTNGSPSTTMMLGGDSAAVAFASSSSSGAGDA
2. Aedae-II   MYLSAGLRKIMDNPPVTSKYQHRSQDEMLLANLNHQQTTNGSPSTTMMLGGDSAAVAFASSSSSGAGDA
3. Aedae-III  MYLSAGLRKIMDNPPVTSKYQHRSQDEMLLANLNHQQTTNGSPSTTMMLGGDSAAVAFASSSSSGAGDA

           70        80        90        100       110       120       130
Consensus  YFRGLAALTGNASGISSMDMSNSTETGIVAPGHTETTVAVIIVYCVLFIIAAGGNLSVVITLFRSRRHR

1. Aedae-I    YFRGLAALTGNASGISSMDMSNSTETGIVAPGHTETTVAVIIVYCVLFIIAAGGNLSVVITLFRSRRHR
2. Aedae-II   YFRGLAALTGNASGISSMDMSNSTETGIVAPGHTETTVAVIIVYCVLFIIAAGGNLSVVITLFRSRRHR
3. Aedae-III  YFRGLAALTGNASGISSMDMSNSTETGIVAPGHTETTVAVIIVYCVLFIIAAGGNLSVVITLFRSRRHR

           140       150       160       170       180       190       200
Consensus  RSRVSLMICHLAVADLMVAFIMIPLEVGWRITVQWHAGNVACKVFLFMRAFCLYLSSNVLVCVSLDRCF

1. Aedae-I    RSRVSLMICHLAVADLMVAFIMIPLEVGWRITVQWHAGNVACKVFLFMRAFCLYLSSNVLVCVSLDRCF
2. Aedae-II   RSRVSLMICHLAVADLMVAFIMIPLEVGWRITVQWHAGNVACKVFLFMRAFCLYLSSNVLVCVSLDRCF
3. Aedae-III  RSRVSLMICHLAVADLMVAFIMIPLEVGWRITVQWHAGNVACKVFLFMRAFCLYLSSNVLVCVSLDRCF

           210       220       230       240       250       260       270
Consensus  AVIYPLRVSAARKRGKIMLGGAWFIAFANAIPQXXXFRVQHHPNVPDFTQCVTFGFFTTPAMETAYNLF

1. Aedae-I    AVIYPLRVSAARKRGKIMLGGAWFIAFANAIPQSIIFRVQHHPNVPDFTQCVTFGFFTTPAMETAYNLF
2. Aedae-II   AVIYPLRVSAARKRGKIMLGGAWFIAFANAIPQSIIFRVQHHPNVPDFTQCVTFGFFTTPAMETAYNLF
3. Aedae-III  AVIYPLRVSAARKRGKIMLGGAWFIAFANAIPQVHV

           280       290       300       310       320       330       340
Consensus  CVVAMYFMPLMVISAAYTVILCEISNRSREKXTXXXXXXXXXXXXXXXXXXXXXXXXXXXXXXXXXXXXX

1. Aedae-I    CVVAMYFMPLMVISAAYTVILCEISNRSREKETSDTSHTGGMRLRCNDLTHIERARQRTLRLTITIVVV
2. Aedae-II   CVVAMYFMPLMVISAAYTVILCEISNRSREKGT-------------------------------------
3. Aedae-III

           350       360       370       380       390       400       410
Consensus  XXXCXTXXXXXXXXXXXXXXXXXXXXXXXXXXXXXXXXXXXXXXXXXXXXXXXXXXXXXXXAPXXXX

1. Aedae-I    FVWCWTPYVVMTLWYMFDRESALKVDGAIQDGLFLMAVSNSCMNPLVYGSYAMKCRRPWRRQMAPNGVQ
2. Aedae-II   ---CLT-------------------------------------------------------AKAPSKWT
3. Aedae-III

           420       430       440       450       460       470       480
Consensus  XPXXXXXXXXXXXXXXXXXXXXXXXXXXXXXXXXXXXXXXXXXXXXXXXXXXXXXRXGSXGRNHVTMA

1. Aedae-I    TPNAAQRRSTDAVSGMVGPHSDRLTGRDNKDELVYEGGGTERNKLKQFGMANGIFARIGSTGRNHVTMA
2. Aedae-II   VP-----------------------------------------------------SRMGSF
3. Aedae-III

           490       500       510       520       530       540       550
Consensus  GGGAAGTSVATTRFGNCTTTVGGRNVTEPIRTVAMSSGSRVKSFQPSYSEFVMMADERSLMLSRPSFYS

1. Aedae-I    GGGAAGTSVATTRFGNCTTTVGGRNVTEPIRTVAMSSGSRVKSFQPSYSEFVMMADERSLMLSRPSFYS
2. Aedae-II
3. Aedae-III

           560       570  577
Consensus  DPTPNEFDSTVDCGLTRSLDTGGWR

1. Aedae-I    DPTPNEFDSTVDCGLTRSLDTGGWR
2. Aedae-II
3. Aedae-III
```

Figure S1. Sequence alignment of *Aedes aegypti* ACP receptor variants I, II, and III. Aligned amino acid sequences of ACPRs from *A. aegypti*, transcript variant I, (GenBank: MF461644), transcript variant II, (GenBank: MF461645), transcript variant III, (GenBank: MF461646). Residues outlined in red indicate predicted transmembrane domains based on the *A. aegypti* sequences. Highlighting of residues indicates % identity with black denoting 100% sequence identity, dark grey denotes 80-100% identity, and light grey represents amino acid positions with 60-80% sequence identity.

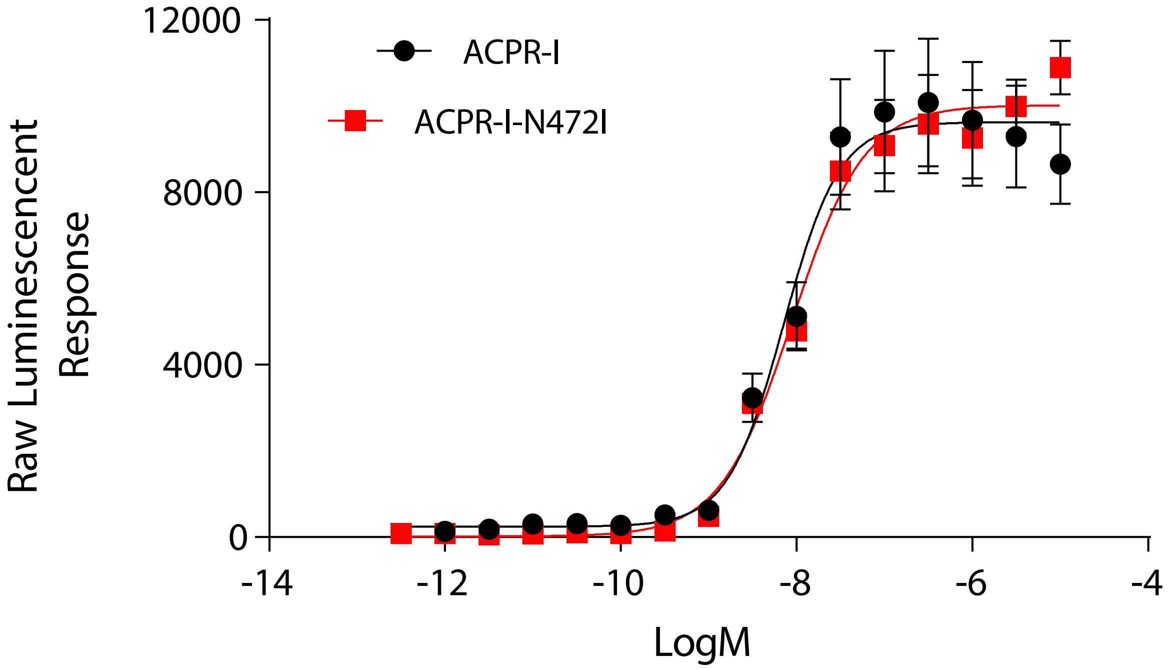

Figure S2. Functional heterologous receptor assay of CHO-K1 aequorin cells transiently expressing the *Aedae*ACPR-I and *Aedae*ACPR-I-N472I. Dose dependent effect on the bioluminescence response (mean 0-15s) after the addition of between 10^{-12} – 10^{-5} M doses of *Aedae*ACP to *Aedae*ACPR-I and *Aedae*ACPR-I-N472I. Luminescence is normalized to the BSA control. Data represent the mean ± standard error (n=4).

Table S1. GenBank accession numbers and references for receptor sequences in Fig. 3.

Receptor	Putative Ligand(s)	Accession Number	Reference(s)
Acyrthosiphon pisum AKHR	AKH	XP_003245941	[1]
Aedes aegypti AKHR1	AKH	XP_001655248	[2]
Aedes albopictus AKHR	AKH	XP_019540948	
Anopheles gambiae AKHR	AKH	ABD60146	[3]
Anopheles darlingi	AKH	ETN67429	[4]
Anopheles sinensis	AKH	KFB53098	[5]
Culex pipiens AKHR	AKH	AAWU01041683	[1]
Apis mellifera AKHR	AKH	NP_001035354	[6]
Bombyx mori AKHR	AKH, ACP	NP_001037049	[7–11]
Musca domestica AKHR		XP_019895730	
Manduca sexta AKHR	AKH	ACE00761	[12]
Drosophila melanogaster AKHR	AKH	AAC61523	[7,13]
Locusta migratoria AKHR		ANW09575	
Polyrhachis vicina AKHR		ADK55068	
Nasonia vitripennis AKHR	AKH	NP_001161243	[14]
Tribolium castaneum AKHR	AKH	NP_001076809	[14]
Periplaneta Americana AKHR	AKH	ABB20590	[14]
Rhodnius prolixus AKHR	AKH	AIJ49751	[15]
Gryllus bimaculatus AKHR	AKH	ADZ17179	[16]
Glossina morsitans AKHR	AKH	AEH25943	
Aedes aegypti CRZR	CRZ	AAGE02020874	[2]
Aedes albopictus CRZR		XP_019554625	
Anopheles gambiae CRZR	CRZ	AAQ67361	[17]
Apis mellifera CRZR	CRZ	NP_001137393	[6]
Culex pipiens CRZR	CRZ	AAWU01031675	[1]
Bombyx mori CRZR	CRZ	NP_001127719	[8,14,18]
Drosophila melanogaster CRZR	CRZ	AAM21341	[19]
Nasonia vitripennis CRZR	CRZ	AAZX01006363	[20]
Bactrocera dorsalis CRZR	CRZ	AQX83392	[21]
Rhodnius prolixus CRZR-A	CRZ	AND99324	[22]
Manduca sexta CRZR	CRZ	AAR14318	[23]
Musca domestica CRZR	CRZ	NP_001295994	
Anopheles gambiae ACPR	ACP	ABX52399	[14]
Anopheles darlingi ACPR	ACP	ETN61096	[24]
Aedes albopictus ACPR	ACP	XP_019559258	
Culex pipiens ACPR	ACP	XP_001842868	[1]
Tribolium castaneum ACPR	ACP	ABX52400	[14]
Nasonia vitripennis ACPR	ACP	NP_001164571	[14]
Bombyx mori ACPR1	ACP, AKH	NP_001127726	[14,23]
Bombyx mori ACPR2	ACP, AKH	NP_001127745	[14,23]
Rhodnius prolixus ACPR-C	ACP, AKH	AKO62858	[25]

References

1. Li, S. *et al.* Adipokinetic hormones and their G protein-coupled receptors emerged in Lophotrochozoa. *Sci Rep* 1–13 (2016). doi:10.1038/srep32789
2. Nene, V. *et al.* Genome Sequence of *Aedes aegypti*, a Major Arbovirus Vector. *Science* **316,** 1718–1723 (2007).
3. Kaufmann, C. & Brown, M. R. Adipokinetic hormones in the African malaria mosquito, *Anopheles gambiae*: Identification and expression of genes for two peptides and a putative receptor. *Insect Biochem Mol Biol* **36,** 466–481 (2006).
4. Mendes, N. D., Freitas, A. T., Vasconcelos, A. T. & Sagot, M.-F. Combination of measures distinguishes pre-miRNAs from other stem-loops in the genome of the newly sequenced *Anopheles darlingi*. *BMC Genomics* **11,** 529 (2010).
5. Zhou, D. *et al.* Genome sequence of *Anopheles sinensis* provides insight into genetics basis of mosquito competence for malaria parasites. *BMC Genomics* **15,** 42 (2014).
6. Elsik, C. G. *et al.* Finding the missing honey bee genes: lessons learned from a genome upgrade. *BMC Genomics* **15,** 86 (2014).
7. Staubli, F. *et al.* Molecular identification of the insect adipokinetic hormone receptors. *Proc Natl Acad Sci USA* **99,** 3446–3451 (2002).
8. Ou, J. *et al.* Transcriptomic analysis of developmental features of *Bombyx mori* wing disc during metamorphosis. *BMC Genomics* **15,** 820 (2014).
9. Suetsugu, Y. *et al.* Large scale full-length cDNA sequencing reveals a unique genomic landscape in a lepidopteran model insect, *Bombyx mori G3 (Bethesda).* **3,** 1481–92 (2013).
10. Huang, H. *et al.* Bombyx adipokinetic hormone receptor activates extracellular signal-regulated kinase 1 and 2 via G protein-dependent PKA and PKC but ß-arrestin-independent pathways. *Biochemistry* **49,** 10862–10872 (2010).
11. Zhu, C. *et al.* Molecular and functional characterization of adipokinetic hormone receptor and its peptide ligands in *Bombyx mori*. *FEBS Lett* **583,** 1463–1468 (2009).
12. Ziegler, R., Isoe, J., Moore, W., Riehle, M. A. & Wells, M. A. The putative AKH receptor of the tobacco hornworm, *Manduca sext*a, and its expression. *J Insect Sci* **11,** 40 (2011).
13. Hauser, F., Søndergaard, L. & Grimmelikhuijzen, C. J. Molecular cloning, genomic organization and developmental regulation of a novel receptor from *Drosophila melanogaster* structurally related to gonadotropin-releasing hormone receptors for vertebrates. *Biochem Biophys Res Commun* **249,** 822–828 (1998).
14. Hansen, K. K. *et al.* Discovery of a novel insect neuropeptide signaling system closely related to the insect adipokinetic hormone and corazonin hormonal systems. *J Biol Chem* **285,** 10736–10747 (2010).
15. Zandawala, M., Hamoudi, Z., Lange, A. B. & Orchard, I. Adipokinetic hormone signalling system in the Chagas disease vector, *Rhodnius prolixus*. *Insect Mol Biol* **24,** 264–276 (2015).
16. Konuma, T., Morooka, N., Nagasawa, H. & Nagata, S. Knockdown of the adipokinetic hormone receptor increases feeding frequency in the two-spotted cricket *Gryllus bimaculatus*. *Endocrinology* **153,** 3111–3122 (2012).
17. Belmont, M., Cazzamali, G., Williamson, M., Hauser, F. & Grimmelikhuijzen, C. J. P. Identification of four evolutionarily related G protein-coupled receptors from the

malaria mosquito *Anopheles gambiae*. *Biochem Biophys Res Commun* **344,** 160–165 (2006).
18. Yamanaka, N. *et al.* Neuropeptide receptor transcriptome reveals unidentified neuroendocrine pathways. *PLoS One* **3,** e3048 (2008).
19. Cazzamali, G., Saxild, N. & Grimmelikhuijzen, C. Molecular cloning and functional expression of a *Drosophila* corazonin receptor. *Biochem Biophys Res Commun* **298,** 31–36 (2002).
20. Werren, J. H. *et al.* Functional and evolutionary insights from the genomes of three parasitoid *Nasonia* species. *Science* **327,** 343–348 (2010).
21. Hou, Q. L. *et al.* A role of corazonin receptor in larval-pupal transition and pupariation in the oriental fruit fly *Bactrocera dorsalis* (Hendel) (Diptera: Tephritidae). *Front Physiol* **8,** 1–14 (2017).
22. Hamoudi, Z., Lange, A. B. & Orchard, I. Identification and characterization of the corazonin receptor and possible physiological roles of the corazonin-signaling pathway in *Rhodnius prolixus*. *Front Neurosci* **10,** 1–12 (2016).
23. Kim, Y.-J. *et al.* Corazonin receptor signaling in ecdysis initiation. *Proc Natl Acad Sci USA* **101,** 6704–6709 (2004).
24. Marinotti, O. *et al.* The genome of *Anopheles darlingi*, the main neotropical malaria vector. *Nucleic Acids Res* **41,** 7387–7400 (2013).
25. Zandawala, M., Haddad, A. S., Hamoudi, Z. & Orchard, I. Identification and characterization of the adipokinetic hormone/corazonin-related peptide signaling system in *Rhodnius prolixus*. *FEBS J* **282,** 3603–3617 (2015).
